# Editorial: Subjective sensations in obesity and related chronic diseases

**DOI:** 10.3389/fnut.2025.1625911

**Published:** 2025-06-06

**Authors:** Xiaohua Wang, Omorogieva Ojo

**Affiliations:** ^1^Department of Cardiology, The First Affiliated Hospital of Soochow University, Suzhou, China; ^2^School of Health Sciences, University of Greenwich, London, United Kingdom

**Keywords:** obesity, chronic conditions, type 2 diabetes, nutrition, appetite, food preferences, exercise

Obesity is a chronic metabolic disease caused by a combination of genetic and environmental factors (1). In 2016, it was reported that about 39% of people were overweight and 13% were obese, globally. The overweight and obese population is projected to account for 57.8% of the world's total population by 2030 (2). Due to the high prevalence of overweight and obesity and the consequent health risks, it has become a major global public health problem. At present, obesity is recognized as one of the important risk factors for chronic diseases such as type 2 diabetes mellitus, cardiovascular diseases, hyperlipidemia, hepatobiliary diseases and certain cancers (3). It has been estimated that obesity is responsible for 44%, 23% and 7–41% of the incidence of diabetes, ischemic heart disease and certain cancers, respectively (4).

One of the causes of the obesity pandemic is the loss of effective appetite control, resulting in distorted energy balance (5). Appetite control operates through two systems, the homeostatic system and the hedonic system (6). The homeostatic system controls energy intake and the hedonic system controls sensory pleasure in eating, and they together regulate hunger and satiety, as well as certain food choices and preferences (7, 8). However, the hedonic system can operate independently of homeostatic signaling when food is very palatable and readily available (9). In addition, obese people display greater food fortification (10) and hedonic hunger (11), a greater liking for sweet flavors (12), higher food cravings (13), and higher energy intake (14) than non-obese people, which may lead to weight gain. Therefore, it is an important entry point for weight reduction by down-regulating the appetite of obese people.

National and international guidelines recommend lifestyle (nutrition and exercise) interventions as the first-line treatment for obesity (15, 16). It is known that ketone bodies produced by the body during a very low-energy diet inhibit the production of growth hormone-releasing peptide, thereby reducing hunger (17). Based on the carbohydrate-insulin model, a low-carbohydrate diet reduces insulin levels and inhibits metabolic fuel deposition into adipose tissue (18). The relative increase in circulating fuels decreases hunger, preference for carbohydrates, and energy intake (19). In addition, a diet rich in unsaturated fatty acids facilitates the production of short-chain fatty acids (SCFAs) (20), of which butyrate binds to G-coupled protein receptor 43 and promotes the secretion of peptide YY (PYY) and glucagon-like peptide-1 (GLP-1) (21), which in turn activates the vagus nerve and the hypothalamus to suppress appetite and eating behavior (22). Meanwhile, dietary fiber is fermented by intestinal microorganisms to produce large amounts of SCFAs (23). Among SCFAs, acetate can activate the citric acid cycle in the hypothalamus and further alter the expression profile of neuropeptides that regulate satiety to suppress appetite (24). In addition, prolonged exercise may increase levels of the satiety hormones, GLP-1 and PYY (25).

This Research Topic, “*Subjective sensations in obesity and related chronic diseases*,” has brought together a Research Topic of insightful studies that delved into various aspects of appetite, food preferences, and their implications for obesity and related chronic diseases.

Within this Research Topic, Al Sabbah et al. provided valuable insights into the relationship between beverage consumption and weight status. Their findings highlight a significant association between the intake of high-sugar beverages (including milk, fruit juice, soft drinks, and energy drinks) and an increased risk of overweight and obesity. Interestingly, milk consumption was specifically linked to obesity and overweight among non-Emirati populations. While overweight and obese students reported preferring fruits and vegetables, they also exhibited a preference for high-calorie, low-nutrient foods. Furthermore, the study identified a significant association between fast food consumption, such as shavarma, and weight gain.

Addressing childhood obesity, Onay et al. explored the family perspective, revealing that parental perception of their child's weight is a significant predictor of body mass index in underweight and overweight children. In the obesity group, in addition to parental perception, factors such as the child's age and gender, fast eating speed, family history of obesity, and parental neglect were also identified as predictive factors. These findings offer a novel perspective for developing binary intervention strategies for childhood obesity.

In the realm of predicting obesity-related chronic diseases, Chen et al. utilized machine learning to identify triglyceride glucose-body mass index (TyG-BMI) as the strongest predictor of osteoporosis in patients with type 2 diabetes mellitus (T2DM). Consistency in the use of TyG-BMI to predict risk of osteoporosis in patients with T2DM was also demonstrated in subgroup analysis, with increase in osteoporosis risk associated with higher TyG-BMI levels. A cross-sectional study by Zhu et al. demonstrated that the Metabolic Score for Visceral Fat (METS-VF) was independently associated with a high incidence of chronic pain, particularly among the elderly population.

Finally, Collet and Schwitzgebel contributed a comprehensive review comparing traditional therapies with the emerging field of precision medicine in rare hereditary obesity. They elaborated on the relevant mechanisms and therapeutic potential of personalized approaches for this specific patient group.

While observational and randomized controlled studies have shed light on the relationship between certain nutrients or exercise and appetite in obese individuals, several questions remain open. The precise interplay between specific nutrients, the optimal mode of combining nutrition and exercise interventions to impact appetite and weight outcomes, and the underlying mechanisms of these effects in obese populations are still areas of active investigation and debate.

In conclusion, this Research Topic offers valuable contributions to our understanding of *Subjective sensations in obesity and related chronic diseases*. The studies presented here underscore the complexity of appetite regulation, highlight the impact of dietary habits and family dynamics, and explore novel predictive markers for obesity-related complications. Future research should continue to explore the intricate mechanisms governing appetite control in obesity, investigate the synergistic effects of combined lifestyle interventions, and further develop personalized approaches for effective obesity management and the prevention of associated chronic diseases.
